# Efficacy and Safety of Ustekinumab in Treatment of Ulcerative Colitis: A Systematic Review

**DOI:** 10.7759/cureus.89108

**Published:** 2025-07-31

**Authors:** Yashasvi Agarwal, Nehal K Bhatt, Samyuktha Harikrishnan, Sanathanan Neelakantan Ramaswamy, Shalvin Chand, Manvitha Bendagiri Matam, Lubna Mohammed

**Affiliations:** 1 Internal Medicine, Jawaharlal Nehru Medical College, Belagavi, IND; 2 Internal Medicine, PramukhSwami Medical College, Anand, IND; 3 Medicine and Surgery, Gulf Medical University, Ajman, ARE; 4 Internal Medicine, Government Erode Medical College and Hospital, Perundurai, IND; 5 Medicine and Surgery, University of Fiji, Lautoka, FJI; 6 Internal Medicine, Gandhi Medical College, Hyderabad, IND; 7 Internal Medicine, Dr V.R.K Women's Medical College, Hyderabad, IND

**Keywords:** monoclonal treatment, remission, systematic review, ulcerative colitis, ustekinumab

## Abstract

Ulcerative colitis (UC) is a chronic, relapsing, and remitting immune-mediated condition requiring long-term therapy. Moderate to severe disease is managed using steroids, sulfasalazine, thiopurines, biologicals [anti-tumor necrosis factor, anti-integrins, and anti-interleukin (IL) 12/23], and small molecules (janus kinase inhibitors, sphingosine-1-receptor modulators). Ustekinumab (UST) is an IgG1 monoclonal antibody acting on IL 12/23 recently authorized to treat moderate to severe UC that is not responsive to other biologic medicines. There remains an unmet need in the management of UC despite the growing availability of therapeutic agents. Current treatment algorithms use a standard approach for all patients, but targeted therapies are required for better outcomes. This systematic review aims to evaluate the safety and efficacy of UST in patients with moderate to severe UC. We also noted the clinical and endoscopic improvement with maintenance of clinical and steroid-free remission across multiple databases to strengthen reproducibility.

This systematic review followed the Preferred Reporting Items for Systematic Review and Meta Analysis (PRISMA) 2020 guidelines. Relevant literature was retrieved from PubMed, PubMed Central, Cochrane Library, Science Direct, and Google Scholar. Articles published in English within the last five years (2020 to 2025) were included. Quality assessment tools were applied to ensure the quality of evidence-based medicine that will be utilized to develop a conclusion and direct future review.

The studies analysed showed a superiority of UST in the induction and maintenance of remission in active, difficult-to-treat UC. Our findings indicate that a reduction in Mayo score with improvement in c-reactive protein (CRP) and fecal calprotectin (fCal) can be used to assess a reduction in inflammatory burden and response to treatment. Histo-endoscopic mucosal healing also provides a long-term clinical assessment of the reduction in disease burden. All safety events that led to drug discontinuation and malignancy were similar for UST therapy and placebo. UST, as a treatment option for moderate to severe UC, can provide an alternative avenue in the development of patient-centric targeted therapies. Further research targets should include the formulation of a standard dosing regimen and the evaluation of the long-term safety profile of the drug. There is also limited literature available for comparative analysis of UST treatment with other available therapeutic options, especially biologic agents, and its effect on extra-intestinal manifestations.

## Introduction and background

Ulcerative Colitis (UC) is a chronic, immune-mediated inflammatory disorder distinguished by periods of relapse and remission, primarily affecting the rectum and colon [1]. In 2023, the global prevalence was estimated to be approximately five million cases, with an increasing incidence [2]. The peak onset age is in the second to fourth decade of life, with 20% of cases of new diagnosis made in individuals older than 60 years of age [2].

It is hypothesized to develop in genetically predisposed individuals after exposure to specific environmental or dietary factors. Its development also involves dysbiosis, dysregulated immunological response, and abnormalities in the epithelial barrier [2]. It has aberrant intestinal inflammation with cytokines, including interleukin (IL) 12/23, implicated in the pathogenesis [3]. 

Intestinal symptoms include bloody diarrhea, abdominal pain, fecal urgency, and tenesmus [4]. Extra-intestinal symptoms can occur in 20 to 35% patients, including primary sclerosing cholangitis, pyoderma gangrenosum, venous thromboembolism, and perianal disease most commonly [2]. No definitive test exists for diagnosis; rather, it relies on an integration of clinical evaluation, inflammatory markers, endoscopic findings, and histological analysis. The severity of inflammation affects the management approach and delivery of therapy [5]. Severity is assessed by the Mayo score, the Lichtiger score mainly for acute severe disease, and the Simple Clinical Colitis Activity Index. The treatment goal is clinical remission, endoscopic and mucosal healing, and normalization of biomarkers, c-reactive protein (CRP) and fecal calprotectin (FCal) [2].

In moderate to severe disease, oral corticosteroids are first-line and superior to sulfasalazine for induction therapy [2]. Maintenance therapy includes immunosuppressants (thiopurines), biologicals (anti-tumor necrosis factor, anti-integrins, and anti-IL 12/23), and small molecules (janus kinase inhibitors, sphingosine-1-receptor modulators) [6, 7]. Some of these agents can lack effectiveness with an elevated risk of infections or cancer after prolonged therapy [8].

Ustekinumab (UST) is an IgG1κ monoclonal antibody targeting the p40 subunit of interleukins 12 and 23, approved for the treatment of moderate to severe UC [9]. Its use has been limited to patients with inadequate response, loss of response, medical contraindications, or intolerance to conventional or other biological therapies [10]. The drug is administered as a one-time 130 mg or 6mg/kg intravenous (IV) infusion for induction, followed by 90 mg subcutaneous (SC) injections every eight to 12 weeks for maintenance therapy. The total annual treatment cost ranges from $20,000 to $30,000, which is similar to the cost range of other biological drugs [11].

The landmark clinical trial conducted by Sands et al. has shown UST's efficacy and safety in causing and sustaining remission in UC. In both the induction and maintenance stages, a decrease in the inflammatory markers, with improvement in the partial and modified Mayo score, was noted. Despite demonstration of histo-endoscopic mucosal healing, there are limited results on long-term clinical outcomes and prevention of colonic carcinoma [12, 13]. Patients who received UST treatment in the trial reported no serious hypersensivity or anaphylaxis reactions. Potential opportunistic infections were reported in four patients, and carcinomas developed in seven patients receiving the drug and one patient in the placebo arm [12]. Long-term safety effects of the drug need further study.

There remains an unmet need in the management of UC despite the growing availability of therapeutic agents. UC is a heterogeneous disease, requiring molecular and individual specifications during management. The current treatment algorithm suggests a standard approach for all patients, whereas adequate treatment requires a targeted approach [14]. Therefore, future studies on biological agents with a focus on targeted therapy for moderate to severe UC are required.

This systematic review identifies the safety and efficacy of UST, a targeted biological treatment option for the management of moderate to severe UC. The review aims to observe the clinical and endoscopic improvement, with reduction in severity index scores after treatment, maintenance of remission, and steroid steroid-free period. This review also aims to provide a transparent methodology with a refined search strategy across multiple databases, thereby improving the reproducibility of findings.

## Review

Methods

This systematic review was conducted in accordance with the Preferred Reporting Items for Systematic Reviews and Meta-Analyses (PRISMA) 2020 guidelines [15]. This systematic review was registered in the International Prospective Register of Systematic Reviews (PROSPERO) under the registration number CRD420251041129.

Database and Search Strategy

A comprehensive literature search was performed to identify relevant studies published between June 1, 2020, to April 30, 2025, through various databases like PubMed, PubMed Central, Cochrane Library, Science Direct, and Google Scholar. Keywords such as Ulcerative Colitis, Ustekinumab, and treatment outcome were utilized to develop a search strategy after being entered into search engines. Additionally, we manually searched for citations. Table 1 provides a more thorough description of the search approach and publications found.

**Table 1:** Summary of Search Strategy from Each Database

Database	Keywords	Search Strategy	Filters	Search Results
PubMed Mesh/Medline/PMC	Ulcerative Colitis, Ustekinumab, Treatment Outcome	Ulcerative Colitis ( "Colitis, Ulcerative/diagnosis"[Mesh] OR "Colitis, Ulcerative/drug therapy"[Mesh] OR "Colitis, Ulcerative/genetics"[Mesh] OR "Colitis, Ulcerative/immunology"[Mesh] OR "Colitis, Ulcerative/mortality"[Mesh] OR "Colitis, Ulcerative/surgery"[Mesh] OR "Colitis, Ulcerative/therapy"[Mesh] ) AND Ustekinumab ( "Ustekinumab/administration and dosage"[Mesh] OR "Ustekinumab/adverse effects"[Mesh] OR "Ustekinumab/drug effects"[Mesh] OR "Ustekinumab/therapeutic use"[Mesh] ) AND "Treatment Outcome"[Mesh]	Last five years, English, Humans, Adults 18+ years	26
PubMed Advanced/Medline/PMC	Ulcerative Colitis, Ustekinumab, Treatment Outcome	((Ulcerative Colitis[Title/Abstract]) AND (Ustekinumab[Title/Abstract])) AND (Treatment Outcome)	Last five years, English, Humans, Adults 18+ years	38
PubMed/Medline/PMC	Ulcerative Colitis, Ustekinumab	(Ulcerative Colitis) AND (Ustekinumab)	Last five years, English, Humans, Adults 18+ years	70
PubMed Central Advanced	Ulcerative Colitis, Ustekinumab, Treatment Outcome	((("colitis, ulcerative"[Mesh Terms] OR Ulcerative Colitis[Abstract]) AND "Ustekinumab"[MeSH Terms]) OR Ustekinumab[Abstract]) AND ("treatment outcome"[All Fields])	Last five years	139
PubMed Central	Ulcerative Colitis, Ustekinumab, Treatment Outcome	"Ulcerative Colitis" AND "Ustekinumab" AND "Treatment Outcome"	Last five years	385
Cochrane	Ulcerative Colitis, Ustekinumab, Treatment Outcome	"ulcerative colitis" in Title Abstract Keyword AND "Ustekinumab" in Title Abstract Keyword AND "Treatment outcome" in All Text	Last five years, English	58
Science Direct	Ulcerative Colitis, Ustekinumab, Treatment Outcome	"Ulcerative Colitis" AND "Ustekinumab" AND "Treatment Outcome"	Last five years, English	146
Google Scholar	Ulcerative Colitis, Ustekinumab, Treatment Outcome	"Ulcerative Colitis" AND "Ustekinumab" AND "Treatment Outcome"	Last five years	396

Inclusion and Exclusion Criteria

Papers released within the previous five years were considered in this systematic review, from June 2020 to April 2025, in English, with full text available, based on human studies and adults above the age of eighteen. We included case reports, observational studies, and randomised control trials, which provided details regarding patients who were diagnosed with UC, moderate to severe intensity, and underwent treatment with UST. Studies published more than five years ago, written in languages other than English, or involving non-human subjects were excluded to ensure relevance and consistency with the objectives. In addition, we excluded studies without full-text availability and grey literature due to limited access to detailed data.

Selection Process

After importing all pertinent articles into Mendeley, duplicates were eliminated. All identified articles were screened by reviewing their titles and abstracts. Relative articles were identified and screened for full-text availability. Relevant literature was assessed according to the inclusion and exclusion criteria. Shortlisted articles that satisfied all the criteria were included in the study.

Risk of Bias Assessment and Process of Data Collection

The chosen studies were subjected to the proper quality assessment instruments based on the study type. We used the Cochrane risk assessment tool (ROB2) for the critical appraisal of randomised control trials (RCT), the risk of bias in non randomised studies of interventions (ROBINS-I) for assessment of observational studies, the Joanna Briggs Institute (JBI) critical appraisal tool for case reports and the risk of bias in systematic reviews (ROBIS) tool for evaluation of systematic reviews and meta-analysis. The primary outcome of the data was focused on the efficacy and safety of UST in the treatment of moderate to severe UC.

Results

**Figure 1:**
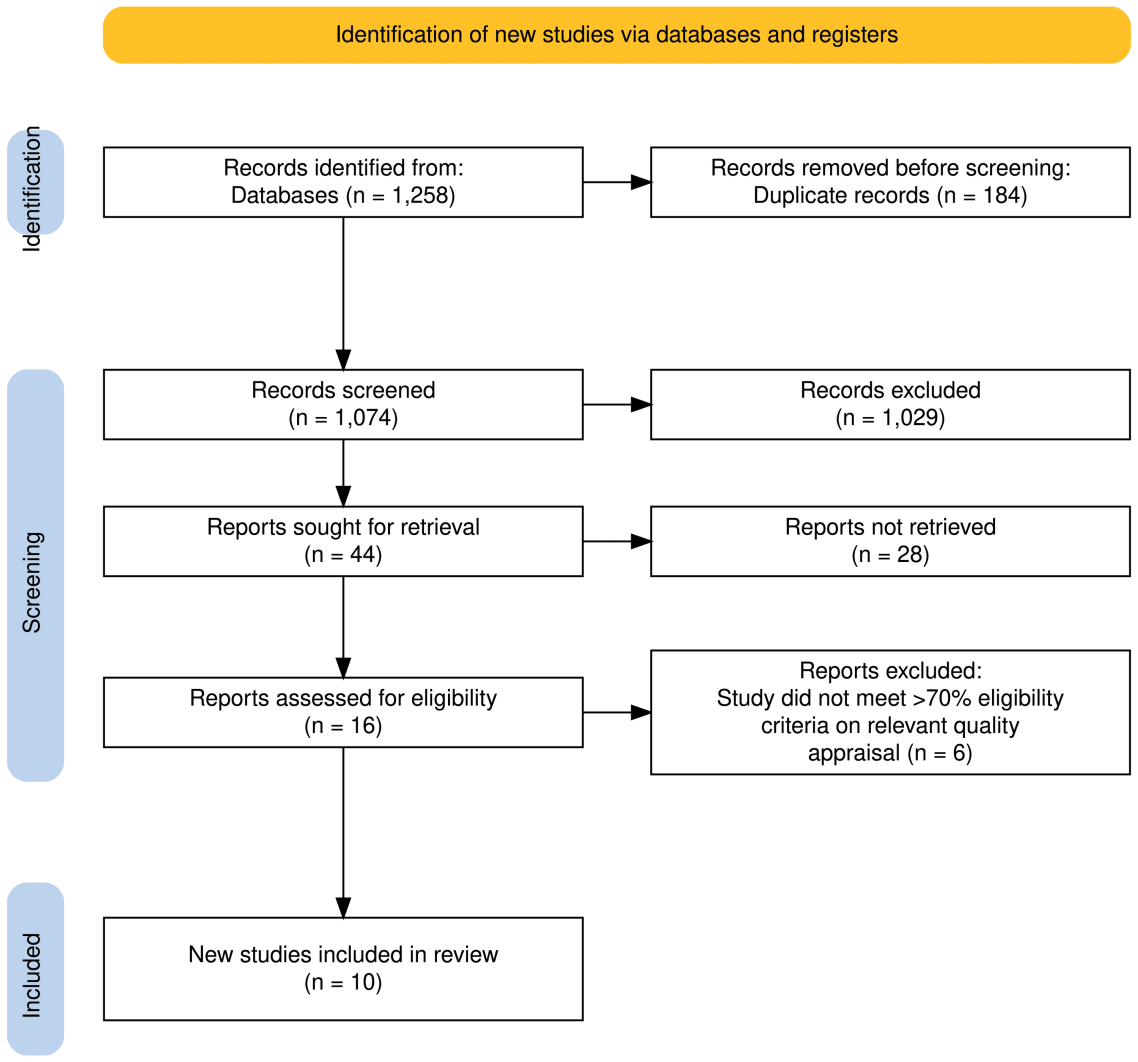
PRISMA Flowchart Illustrating the Article Screening and Selection Process

A total of 1,258 articles were initially retrieved across the selected databases. Duplicate entries (n = 184) were identified and removed using Mendeley reference manager. This resulted in 1,074 unique records, which were screened based on titles, abstracts, and availability of full-text articles. Following this, 44 full-text articles were selected for further review, and 1,029 articles were excluded. Among the 44 full-text articles assessed, 28 were excluded based on predefined inclusion and exclusion criteria. The remaining 16 articles underwent eligibility screening and quality assessment. Based on this evaluation, 10 high-quality articles were selected for inclusion in the final systematic review. The PRISMA 2020 flow diagram illustrating this selection process is presented in Figure 1.

Quality Appraisal

All 16 eligible articles were evaluated using standardised quality appraisal tools appropriate to their study design: ROBINS-I for observational studies, ROB2 for RCTs, ROBIS for systematic reviews and meta-analyses, and JBI critical appraisal checklist for case reports. Studies were categorised as high-quality (>70%), moderate-quality (50-70%), or low-quality (<50%). Only high-quality studies were included in the final analysis. Details of each study’s quality appraisal are shown in Table 2, while individual scoring breakdowns are available in Appendices 1, 2, 3, 4.

**Table 2:** Comprehensive Examination of the Quality Assessment Procedure as well as the Techniques and Resources Employed to Evaluate the Reviewed Article ROBINS-I: risk of bias in non randomised studies of interventions, ROB2: Cochrane risk assessment tool, RCT: randomised control trial, ROBIS: risk of bias in systematic reviews, JBI: Joanna Briggs Institute.

Serial Number	Study Name	Type of Study	Quality Assessment Tool	Score (Acceptable score >70%)
1	Sabhan et al. [16]	Observational Studies	ROBINS-I	85.71%
2	Molander et al. [17]	Observational Studies	ROBINS-I	85.71%
3	Amiot et al. [8]	Observational Studies	ROBINS-I	85.71%
4	Chaparro et al. [18]	Observational Studies	ROBINS-I	85.71%
5	Danese et al. [3]	RCT	ROB2	90%
6	Abreu et al. [19]	RCT	ROB2	90%
7	Afif et al. [13]	RCT	ROB2	90%
8	Li et al. [20]	RCT	ROB2	90%
9	Uchida et al. [9]	Systematic Review and Meta-analysis	ROBIS	100%
10	Affendi et al. [21]	Case Report	JBI Critical Appraisal Tool	93.75%

Study Characteristics

Following the selection and quality assessment procedure, 10 pertinent papers were included. All four retrospective observational studies [8, 16-18], four RCTs [3, 13, 19, 20], one systematic review [9], and one case report [21] were classified as having high quality. We analysed all the articles comparing the inclusion criteria, sample size, age of patients in the study, average duration of disease, disease severity, and prior treatment history. The dose and schedule of UST received, with safety data on the drug and conclusion of the study, were analysed. Table 3 below describes the articles retained in our study.

**Table 3:** Study Characteristics and Outcomes 5-ASA: 5-Aminosalicylic Acid, Anti-TNF: Anti-Tumour Necrosis Factor, IV: Intravenous, JAK inhibitors: Janus Kinase Inhibitors, RCT: Randomized Controlled Trial, SC: Subcutaneous, UC: Ulcerative Colitis, UNIFI: Ustekinumab as Induction and Maintenance Therapy for Ulcerative Colitis, UST: Ustekinumab. Dosing units vary between the studies that have been conducted

Author, year	Type of study	Inclusion criteria	Sample size	Age, years	Disease duration, years	Severity of disease	Prior treatment	UST dosage	Conclusion
Molander et al. [17]	Retrospective observational study	Adults 18+ years, on UST therapy for UC between 1 September 2019 to 31 December 2021 in one of 16 participating Finnish hospitals. Failed prior therapy (mesalamine, thiopurines, biologicals, JAK inhibitors). History of pan-proctocolectomy, ileoanal anastomosis, ileostomy, and concurrent diagnosis of Crohn’s disease was excluded.	221	40.3	7.6	Severe in >91% patients	Thiopurines 23%, 5-aminosalicylic acid 56%, budesonide 5%, oral steroids 52%, IV steroids 5%, adalimumab 39%, infliximab 79%, vedolizumab 48%, golimumab 10%, tofacitinib 18% and others <3%.	Induction dose IV 390mg in 53% patients, 520mg in 40% and either modified dose or 260mg in 7% patients. Maintenance dose SC was given to 91% patients. 90mg every 8 weeks in 81% patients, 90mg every 12 weeks in 9% patients, and 90mg every 6 weeks in 8% patients.	UST has sustained clinical effectiveness and reduces steroid dependence. More frequent UST dosing is recommended.
Sabhan et al. [16]	Observational study	Adults 18+ years received at least 1 UST dose since 2019 with severe disease and brought into remission with prior biologicals but developed antibodies. Prior UST exposure and planned cessation of therapy in 12 months were excluded.	96	36	11 (based on calculation from disease onset, average age at 25 years).	Moderate to severe, with 96% having active disease	Thiopurines 14%, oral steroids 36%, local steroids 15%, oral mesalazine 58%, local mesalazine 14%, treatment failure with biologicals ≥1 in 98%, ≥2 in 61% and ≥3 in 35% patients.	Induction dose IV based on standard protocols, exact dose not mentioned. Maintenance dose SC based on standard protocols, 8-week schedule in 53 patients, 12-week schedule in 30 patients, 4- and 6-week schedule in 1 patient each. Exact dose not mentioned.	UST is an effective biological drug in difficult-to-treat patients. It has a desirable safety profile and remission rate with reduced steroid use after 12 months.
Chaparro et al. [18]	Observational study	Adults 18+ years, who, at least 16 weeks before the data analysis for active UC, had at least one IV dose of UST, with a partial Mayo score of ≥2. Patients who received UST for indications other than UC or who had colonic resection were excluded.	95	47	-	Moderate to severe, median partial Mayo score 6.	Concomitant immunosuppression in 18%, steroids in 58%, anti-TNF agents in 98%, vedolizumab in 86%, and tofacitinib in 31% patients.	Induction dose IV 6mg/kg, 91 patients received a second dose between 6 and 12 weeks, and 84 patients received a third dose between 12 and 20 weeks. Maintenance dose SC based on standard protocols, 8-week schedule in 24 patients, 12-week schedule in 3 patients, 4- to 6-week schedule in 3 patients. Exact dose not mentioned.	UST is effective in inducing remission even in a highly refractory population. Patients with high inflammatory burden have a lower chance of achieving short-term remission.
Amiot et al. [8]	Observational study	Adults 18+ years, on UST therapy for UC between January 2019 to September 2019. Patients who had partial or total colectomy or were initiated on UST for extra-intestinal manifestations were excluded.	103	39.3	7.6	Moderate to severe disease with a mean total Mayo clinical score of 8.5.	Immunosuppressants in 84.5%, one anti-TNF agent in 29.1%, ≥2 anti-TNF agents in 69.9%, vedolizumab in 85.4% and tofacitinib in 9.7% patients. Concomitant treatment with steroids in 39.8%, immunosuppressants in 14.9% and both in 8.7% patients.	Induction dose 6mg/kg IV. Maintenance dose 90mg SC every 8 to 12 weeks. Dose was intensified to every 4 to 8 weeks in insufficient responders.	For patients with refractory UC, UST effectively induces steroid-free clinical remission. Clinical severity with the use of anti-TNF and vedolizumab agents is associated with a reduced probability of steroid-free clinical remission.
Afif et al. [13]	Randomized Control Trial (RCT)	Adults 18+ years, with an established diagnosis of moderate to severe UC. Poor response or adverse consequences from anti-TNF agents, vedolizumab, or non-biologicals. They should have completed the induction and maintenance phase of the UNIFI study to be included in the long-term extension phase.	399	41.6	7	Moderate to severe disease with a mean Mayo score of 9.	Immunomodulators, steroids, biological naïve patients, and a history of at least 1 biological drug failure, including anti-TNF agents and vedolizumab. All patients received induction UST therapy.	Maintenance dose 90mg SC at 8- and 12-week intervals, and a placebo group. Dose adjustment based on clinical judgement from week 56 for randomized patients in long-term extension till week 188.	UST treatment in moderate to severe UC responded well to IV induction and SC maintenance therapy through 4 years of treatment. The tolerability characteristics were consistent with known adverse effects.
Danese et al. [3]	Randomized Control Trial (RCT)	Adults 18+ years, with an established diagnosis of moderate to severe UC at least 3 months before screening. They were inadequate responders or intolerant to conventional and biological therapy, or naïve to biological therapy.	961	41.7	7	Moderate to severe disease with a mean Mayo score of 9	Immunomodulator use in 26.9%, steroids in 31.6%, anti-TNF agents in 48.6%, vedolizumab in 16.8% and biological naïve in 41.3% patients.	Induction dose IV is 6mg/kg, 130mg, and placebo, randomized to 3 groups. At week 8, patients in the placebo group were evaluated and included in the extended induction group to receive IV 6mg/kg. Responders at weeks 8 or 16 were included in the maintenance group, 90mg SC at 8- and 12-week intervals, and the placebo group.	UST induces rapid clinical response and remission with improvement in symptoms within 7 days. There is a reduction in inflammatory burden within 2 weeks of treatment. Previous exposure to biological agents do not affect results.
Abreu et al. [19]	Randomized Control Trial (RCT)	Adults 18+ years, with an established diagnosis of moderate to severe UC. Poor response or adverse consequences from anti-TNF agents, vedolizumab, or non-biologicals. They should have completed the induction and maintenance phase of the UNIFI study to be included in the long-term extension phase.	399	41.4	7.4	Mild to moderate with partial Mayo score 2.5 after induction therapy	Biological failure in 44.1%, anti-TNF agents in 32.1%, vedolizumab in 12%, and both in 11.8% patients. Steroids and immunomodulators were continued when needed. All patients received induction UST therapy.	Maintenance dose 90mg SC at 8- and 12-week intervals, and a placebo group. Dose adjustment based on clinical judgement from week 56 for randomized patients in long-term extension.	UST therapy for moderate to severe active UC was effective for maintenance therapy through the third year. Safety profile in the third year showed no new adverse events.
Li et al. [20]	Randomized Control Trial (RCT)	Adults 18+ years, with a confirmed diagnosis of moderate to severe UC. Poor response or adverse consequences from anti-TNF agents, vedolizumab, or non-biologicals. Previous treatment with IL-12/23 antagonists, imminent colectomy, cancer, or active infections were excluded.	885, with a total of 2,630 biopsy samples analyzed.	41.4	8.1	Moderate to severe disease with a mean Mayo score of 8.8	Immunomodulator use in 26.9%, steroids in 31.6%, anti-TNF agents in 48.6%, vedolizumab in 16.8% and biological naïve in 41.3% patients	Induction dose IV is 6mg/kg, 130mg, and placebo, randomized to 3 groups. Those in the placebo group were offered an induction dose IV 6mg/kg after completion of the induction phase. Responders in the induction group at week 8 and 16 were randomized to receive a maintenance dose of 90mg SC at 8- and 12-week intervals, and the placebo group	The outcome of histo-endoscopic mucosal healing gives additional information regarding clinical status for UST treatment in UC. More research is needed to confirm the prediction of remission and its use in daily practice
Uchida et al. [9]	Systematic Review and Meta-analysis	Prospective and retrospective observational cohort studies, adults 18+ years. Those receiving drugs for only maintenance and a history of colectomy were excluded.	851 patients, 14 studies	41.1, available for 13 studies.	8.05, available for 8 studies.	Mild 3 studies, moderate 9 studies, not reported 2 studies.	Prior immunomodulator 6 studies, steroids 10 studies, anti-TNFa11 studies, vedolizumab 9 studies, tofacitinib 5 studies.	Induction dose IV 6mg/kg in 4 studies, weight-based 260mg <55kg, 390mg 55 to 85kg, 520mg >85kg in 1 study, maintenance dose 90mg SC every 8 to 12 weeks in 6 studies.	UST is safe and effective for UC. Early use, prior to other biological agents may be effective to maximize drug potential.
Affendi et al. [21]	Case Report	Comorbidities include ischemic heart disease, congestive heart failure, diabetes, and chronic obstructive pulmonary disease	1	75	1 month of active symptoms.	Severe	IV and oral steroids, mesalamine.	Induction dose 390mg IV, maintenance dose 90mg SC at week 2, week 6, and every 8 weeks.	Clinical remission, repeat colonoscopy at week 24 shows complete mucosal healing.

Clinical remission rates were compared across the nine studies [3, 8, 9, 13, 16-20] included. We did not include the case report in this analysis, as the patient achieved clinical remission after treatment, without an available rate. Clinical remission and steroid-free clinical remission were defined differently by each study; the time interval of analysis was also variable and should be considered when interpreting the results. Table 4 below shows the remission rates in the studies included. Each study demonstrated the sustained effectiveness of UST, showing a reduction in UC severity and a decrease in reliance on steroid use.

**Table 4:** Clinical Remission and Steroid Free Clinical Remission rates Dosing units vary between the studies that have been conducted. The definitions for clinical-remission and steroid-free clinical remission, and time interval of data analysis are not standardized

Study Name	Clinical Remission	Steroid-Free Clinical Remission
Molander et al. [17]	Baseline: 12%	Baseline: 7%
	16 weeks: 49%	16 weeks: 93%
	6 months: 55%	6 months: 84%
	12 months: 68%	12 months: 92% Percent of steroid-free remission from those who have already achieved clinical remission
Sabhan et al. [16]	3 months: 9 of 71 (12.68%) patients	3 months: 9.4%
	6 months: 25 of 42 (59.52%) patients	-
	12 months: 28 of 37 (75.68%) patients	12 months: 29.2%
Chaparro et al. [18]	16 weeks: 35%	-
	24 weeks: 39%	24 weeks: 30%
	52 weeks: 33%	52 weeks: 32%
Amiot et al. [8]	8 weeks: 16% in patients receiving 130mg and 6mg/kg dose, 5% in placebo	-
	12 to 16 weeks: 39.8%	12 to 16 weeks: 35%
	44 weeks: 38% in the 12-weekly dose interval group, 44% in the 8-weekly dose interval group, 24% in the placebo group.	-
Afif et al. [13]	200 weeks: 55.2%	200 weeks: 53.2%, from those who have already achieved clinical remission
	55.8% in the 12-weekly dose interval group	96.8% in the 12-weekly dose interval group
	54.5% in the 8-weekly dose interval group	94.8% in the 8-weekly dose interval group
Danese et al. [3]	44 weeks: 37.7% for those who received 130mg as an induction dose	-
	48.9% for those who received 6mg/kg as an induction dose	-
Abreu et al. [19]	92 weeks: 64.5% in the 12-weekly dose interval group, 67.8% in the 8-weekly dose interval group	92 weeks: 61.6% in the 12-weekly dose interval group, 65.9% in the 8-weekly dose interval group
	152 weeks: 54.1% in the 12-weekly dose interval group, 56.3% in the 8-weekly dose interval group	152 weeks: 51.2% in the 12-weekly dose interval group, 55.1% in the 8-weekly dose interval group
Li et al. [20]	8 weeks: 39.3% for those who received 130mg as an induction dose, 34.8% for those who received 6mg/kg as an induction dose, 21.6% in the placebo group	-
	44 weeks: 57.6% in the 12-weekly dose interval group, 60.8% in the 8-weekly dose interval group, 32.3% in the placebo group	-
Uchida et al. [9]	12 studies evaluated	11 studies evaluated
	8 weeks: 55%	-
	16 weeks: 36.1%	16 weeks: 29.7%
	6 months: 46.6%	6 months: 30.1%
	12 months: 38.6%	12 months: 38.8%

The safety profile of each study, including the number or percent of patients affected, has been summarized in Table 5 below. Adverse events noted were relatively rare, most commonly comprising infections, including pneumonia, nasopharyngitis, upper respiratory tract infections, and arthralgia. Malignancy was noted in the long-term extension studies, but the significance and relation to treatment have yet to be established.

**Table 5:** Side Effects of UST Reported in Included Studies UC: Ulcerative Colitis, CMV: Cytomegalovirus.

Study Name	Side Effects	Number of Patients Reported
Molander et al. [17]	Discontinuation due to adverse effects – no details available	<6%
	Antibody formation	<6%
Sabhan et al. [16]	Primary non-responders	20%
	Adverse events or intolerance to drug – No serious infections, malignancy, or death reported	8%
	Loss of response	6%
	Need for surgery	2%
Chaparro et al. [18]	Dry skin and itching, mild and did not cause drug discontinuation	1
	Pneumonia did not cause drug discontinuation	1
	SARS-CoV-2 pneumonia and death after 43 weeks of drug treatment	1
Amiot et al. [8]	Arthralgia	1
	Inflammatory bowel disease exacerbation	3
	Pneumonia	1
	Dental abscess	1
	Skin rash	1
	Symptomatic urolithiasis	1
	Serious adverse event – UC exacerbation, pneumonia leading to hospitalization	4 – 3,1
Afif et al. [13]	Nasopharyngitis	16.82/ 100 PY
	UC worsening	15.04/100 PY
	Upper respiratory tract infections	5.35/100 PY
	Non-melanoma skin cancers	0.54/100 PY
	Opportunistic infections – CMV, Listeria monocytogenes, oral herpes with neutropenia	4 – 2, 1, 1
	Colorectal carcinoma	1
	Rectal carcinoma	1
	Death due to cardiac arrest (after 1 maintenance dose)	1
Danese et al. [3]	Upper respiratory tract infections, headache, arthralgia, nasopharyngitis occurred most, with rates similar to the placebo group. No deaths or malignancies reported.	-
Abreu et al. [19]	Adverse effects – nasopharyngitis, upper respiratory tract infections, UC worsening	244.41/100 PY
	Serious adverse effects	8.01/100 PY
	Non-melanoma skin cancer	0.73/100 PY
	Neutropenic sepsis with oral herpes simplex infection	1
	Death due to cardiac arrest	1
Li et al. [20]	-	-
Uchida et al. [9]	Any adverse events	10.9%
	Infections – pneumonia, dental abscess, Clostridium difficile, urinary tract infection, pharyngitis, otitis media, COVID-19	4.3% - 1, 2, 2, 1, 2, 1, 1
	Breast carcinoma	0.4%
	Skin rash	1.6%
	Arthralgia	1.9%
	Inflammatory bowel disease exacerbation	3.9%
	Symptomatic urolithiasis	0.4%
	Gastroenteritis	0.8%
	Myocardial infarction	0.4%
	Fatigue	0.4%
	Rectal adenoma	0.4%
	Hearing loss	0.4%
	Atrial fibrillation	0.4%
	Pituitary adenoma	0.4%
	Retinal detachment	0.4%
Affendi et al. [21]	No significant adverse effects noted	1

Discussion

Evaluation of treatment in a real-world setting, where disease characteristics are complicated and landmark RCTs are analysed, has given a comprehensive approach to the use of UST in UC. The result of this review can provide a valuable approach to patient care in routine clinical practice.

Pathogenesis and Histological Assessment

Ulcerative colitis (UC) was first described by Samuel Wilks in 1859. Although the exact pathogenesis remains incompletely understood, a combination of genetic susceptibility, environmental triggers, epithelial barrier dysfunction, dysbiosis, and immune dysregulation has been implicated [4]. Microscopic inflammation is independently associated with colorectal cancer risk and clinical relapse, even in patients with endoscopically normal mucosa [20].

Colonic mucosal injury induces inflammation through upregulation of tumor necrosis factor-alpha (TNF-α) and IL production, leading to local immune cell infiltration-primarily neutrophils and mononuclear cells. This results in crypt architectural distortion, crypt abscesses, and epithelial damage [22]. Pavlidis et al. noted that overexpression of IL-23 contributes to multisystem inflammation, making it a key therapeutic target. Preclinical studies have demonstrated that neutralizing or deleting the IL-23 p19 subunit, the principal target of ustekinumab (UST), significantly reduces colitis severity [23]. A recent study also proposed that complete resolution of neutrophil-associated inflammation should be considered a treatment goal in UC [24].

In a study by Li et al., patients who achieved clinical remission following eight to 16 weeks of UST induction therapy demonstrated over 80% histological improvement. This was defined by neutrophil presence in fewer than 5% of crypts, with no crypt damage, ulceration, or granulation tissue. The study concluded that histo-endoscopic mucosal healing is a stronger predictor of favorable long-term outcomes than histology or endoscopy alone [20].

Another study conducted by Amiot et al. evaluated endoscopic activity through week 12 to 16 using the Ulcerative Colitis Endoscopic Index of Severity (UCEIS) and Mayo Clinic endoscopic sub-score. There was a notable decrease in the UCEIS score from 5.0 ± 1.2 to 3.8 ± 1.9 and a decrease in the Mayo Clinic endoscopic sub-score from 2.7 ± 0.5 to 2.2 ±1.0. 16.3% patients had a UCEIS score of 0-1, and 18.4% patients had a Mayo Clinic endoscopic sub-score of 0-1. Those with steroid-free clinical remission had a reduction in UCEIS score from 4.7 ± 1.3 to 1.8 ± 1.4 and a reduction in the Mayo Clinic endoscopic sub-score from 2.5 ± 0.5 to 1.5 ± 1.1. They concluded that endoscopic improvement should be a therapeutic target with better long-term outcomes and mucosal healing in UC [8].

Assessment of Disease Severity

Clinical trials use the Mayo score as a composite measure of disease activity. The modified Mayo score (mMayo) is currently used to assess UC disease severity in clinical practice. It includes evaluation of rectal bleeding, stool frequency, and endoscopic appearance of colonic mucosa, with a maximum score of 9. The Mayo score had an additional component of Physician’s Global Assessment (PGA), which is no longer included in the mMayo. The partial Mayo Score (pMayo) was developed to include stool frequency, rectal bleeding, and PGA, as endoscopy is not performed at every clinical visit [25].

In a study executed by Danese et al., the changes in pMayo were evaluated from baseline through week two, four, eight, and 16 after receiving an induction dose of UST 130mg and 6mg/kg. There was a decrease by 1.5 at week two, 2.1 at week four, 2.6 at week eight, and 3.7 at week 16 in those receiving the 130mg dose. The reduction in those receiving a 6mg/kg dose was 1.6 at week two, 2.5 at week four, 2.9 at week eight, and 3.6 at week 16. Both UST induction groups showed significantly better results compared to the placebo group, providing a rationale for treatment with the drug [3].

Another study conducted by Afif et al. evaluated 213 patients during long-term extension treatment with UST at 200 weeks. The Mayo score clinical remission was seen in 70.1% who received 12 weekly doses and 69% who received 8 weekly doses, mMayo response was seen in 93.1% who received 12 weekly doses and 97.6% who received 8 weekly doses and endoscopic improvement was seen in 81.6% who received 12 weekly doses and 79.8% who received 8 weekly doses [13]. This highlighted the long-term impact of maintenance treatment and the difference in dosing schedule.

Amiot et al. also showed that the pMayo decreased by 2.3 ± 2.7 points, where rectal bleeding and stool frequency sub-scores of 0 were noted in 19.4% patients after 12 to 16 weeks of treatment with UST. The Selecting Therapeutic Targets in Inflammatory Bowel Disease (STRIDE) committee stated that normalizing bowel movements and stopping rectal bleeding should be the goals of treatment of UC, which is met by UST, as reflected by the reduction in Mayo score [8].

Biomarkers for Assessment of Severity

Endoscopic examination rates are generally lower in clinical practice due to patient preference. Therefore, alternatives to endoscopic examination, such as CRP and FCal are commonly utilized in clinical settings to evaluate disease activity [16]. The STRIDE II committee also endorsed biomarkers like CRP and FCal to measure disease activity, inflammatory burden of disease, and response to treatment [3].

A randomized control trial conducted by Danese et al. evaluated CRP and FCal at weeks two, four, eight, and 16 of treatment. From week two through eight, the decline in CRP levels was noticeably larger in patients treated with both 130mg and 6mg/kg doses of UST vs the placebo group. Normalization of CRP levels at the end of 16 weeks was seen in 43.2% patients receiving 130mg vs 42.7% patients receiving 6mg/kg of drug, demonstrating a significant reduction in inflammatory burden. FCal levels were significantly reduced by week four in patients receiving both induction doses vs placebo treatment. Normalization of levels was reported in 30.7% patients receiving a 130mg dose and 28.5% patients on a 6mg/kg dose at the end of 16 weeks of treatment [3].

Studies carried out by Molander et al. and Sabhan et al. showed a significant reduction in FCal levels. They reported a reduction from 1262mg/dL at baseline to 662mg/dL at 16 weeks and 215mg/dL at 12 months follow-up, and 864mg/dL at baseline to 178mg/dL at 12 months, respectively. Demonstrating that improvement in FCal during treatment with UST is a reliable indicator of drug effectiveness, intestinal mucosal healing, and can be used as a surrogate marker for endoscopic remission [16, 17].

Long-term maintenance studies led by Abreu et al. and Afif et al. suggested that median serum CRP levels and FCal concentrations remained low from week 44 through weeks 152 and 200, respectively. This indicated sustained effectiveness in the reduction of inflammatory burden with continued UST treatment [13, 19].

Management of Ulcerative Colitis

The primary treatment objective in individuals with UC is to achieve and sustain long-term remission, given its relapsing and remitting nature [12]. Key considerations include the medication’s safety, tolerability, and ability to induce and maintain clinical remission [19]. In mild to moderate UC, up to 40% patients' responses to first-line treatment options are insufficient or nonexistent, such as 5-aminosalicylic acids (5-ASAs), steroids, and immunomodulators. For those with moderate to severe UC who are refractory to conventional options, biological therapies and Janus kinase (JAK) inhibitors are used for induction and maintenance of remission [17].

The growing risk of infections, cancer, and limited efficacy of existing treatments has led to a rising demand for new therapies that act through alternative mechanisms [8]. Maintaining a sustained response to biologic therapy is essential in the management of UC, due to the need of extended treatment. However, approximately 30-40% of patients stop biologic therapy because of adverse effects or insufficient clinical improvement [19]. Direct, head-to-head comparisons between UST and other first-line or biologic therapies remain lacking. Current treatment algorithms largely adopt a standardised approach, although optimal care may require individualised, patient-tailored strategies [14]. Therefore, future studies on tailored therapy for patient-centric management remain an unmet need.

Studies performed by Sabhan et al., Chaparro et al. and Danese et al. suggested that whether the patients were biologic naïve, poor responders, or intolerant to biologic therapy, the degree of improvement in symptoms, clinical ratings, and biomarker levels remained consistent, thus supporting benefit of UST therapy in difficult cases [3, 16, 18]. On the contrary, studies performed by Amiot et al., Afif et al., and Abreu et al. suggested that patients who were new to biologics showed better efficacy results than those who had previously experienced biologic failure. Reduced likelihood of steroid-free clinical remission was observed after UST treatment in those who had received anti-TNF agents and vedolizumab treatment [8, 13, 19]. The influence of previous therapies on UST efficacy in UC remains poorly understood [9].

Abreu et al. observed that the durability of anti-TNF therapies was reduced in the absence of concomitant immunosuppressants such as thiopurines or methotrexate. In contrast, UST demonstrated sustained efficacy even as monotherapy [19]. Sabhan et. al. showed 20 patients who discontinued UST after 12 months of treatment, of which 35% were treated with Vedolizumab, 25% with Infliximab, 25% with Adalimumab, 10% with Certolizumab, and 5% with Golimumab [16]. The complex interplay between biologic drugs requires further analysis.

Ustekinumab Treatment

Ustekinumab (UST) is a monoclonal antibody that targets the p40 subunit of interleukin (IL)-12/23. It has been approved for the treatment of Crohn’s disease, psoriasis, and psoriatic arthritis. The pivotal Ustekinumab as Induction and Maintenance Therapy for Ulcerative Colitis (UNIFI) clinical trial, which included 961 patients, demonstrated that clinical remission at week eight was significantly higher among patients receiving UST (either 130 mg or 6 mg/kg IV, both 16%) compared to placebo (5%). By week 44, sustained clinical remission was also superior in patients receiving subcutaneous (SC) UST every eight weeks (44%) or every 12 weeks (38%) compared to placebo (24%) [12].

Danese et al. further reported that UST significantly improved two core UC symptoms-rectal bleeding and increased stool frequency-over the first 16 weeks of treatment [3]. Similarly, Molander et al. observed marked reductions in clinical severity and inflammatory biomarkers within 16 weeks of UST initiation [17]. Afif et al. also showed long-term SC treatment to be effective and safe in maintaining remission through four years of treatment after IV induction therapy. Long-term treatment participants typically experience superior safety and efficacy results than those who stop [13]. 

Dosing and frequency varied across studies, as noted in Table 3 above. Danese et al. showed superior remission rates at week 44 with a 6 mg/kg IV induction dose compared to 130 mg [3]. During the maintenance phase, most patients received SC UST every eight weeks, as noted by Sabhan et al., who found no added benefit with more frequent dosing [16]. However, another study conducted by Amiot et al. reported, half of the patients required dose escalation to enhance their initial response to UST induction therapy in some retrospective reports [8]. UST dose intensification has been useful for remission in Crohn’s disease; however, data in UC are deficient. Additional exploration is necessary to define the role of dose intensification in UC [18].

Research done by Molander et al. and Afif et al. noted UST to be well tolerated in UC. Safety events, which led to drug discontinuation and malignancy, were at rates not greater than placebo [13]. The rate of adverse events decreased with additional UST exposure according to Abreu et.al. They proposed it to be due to patient discontinuation from the study, patient fatigue in reporting adverse events, or sustained efficacy of the drug [19]. Chaparro et. al. noted 9.5% patients needed to undergo colectomy due to UST failure, 14 weeks after starting the drug [18]. Table 5 above has evaluated the safety profile of all studies included. Long-term data on the drug safety profile needs further analysis.

Limitations

Our study had several limitations. We included data from studies in the past five years, which were published in English and had free full text available, thus limiting the number of articles screened. Children below 18 years were excluded, and the geriatric population was not adequately represented, thus creating a lacuna in the study population. The dosing regimen of UST, severity of disease, definitions of clinical remission and steroid-free clinical remission, and time points at which efficacy of UST was evaluated varied across the studies, without a generalization criterion. This could contribute to data heterogeneity and reliability. The retrospective observational studies included could have recall, selection, and information bias introduced. Some randomized controlled trials (RCTs) in the maintenance phase involved unblinding and selective continuation of patients who initially responded to therapy, which may limit the generalisability of findings. All studies included did not conduct regular colonoscopies and histological analysis, thus limiting the data available for results and their incorporation into clinical practice. There are no studies available that compare UST effectiveness to other treatment options, including biologic therapies, thus limiting data to formulate a treatment algorithm. The effect of treatment on extra-intestinal manifestations of UC, which contribute significantly to disease morbidity, also remains an area yet to be explored.

## Conclusions

Clinical evidence supports the safety and efficacy of UST for patients with moderate-to-severe, difficult-to-treat UC. Optimal long-term outcomes may be achieved by integrating histo-endoscopic mucosal healing as a therapeutic target, given its strong predictive value for sustained remission. In routine clinical practice, non-invasive inflammatory biomarkers such as CRP and FCal are valuable for monitoring disease activity and therapeutic response, while regular assessment of the Mayo score further guides clinical management. Further investigations are necessary to define optimal dosing strategies and administration schedules for UST, to evaluate its impact on UC-related complications and extra-intestinal manifestations, and to comprehensively assess its long-term safety profile. Comparative studies evaluating the efficacy of UST in relation to other biological agents, as well as investigations into the outcomes of combination drug regimens, are also needed. The heterogeneity of UC pathogenesis, which gives rise to diverse intestinal and extra-intestinal manifestations, underscores the importance of a personalized, patient-centric treatment approach for optimizing therapeutic outcomes and maintaining long-term remission. Importantly, the role of UST in managing extra-intestinal manifestations of UC remains inadequately explored. Therefore, future research on biological agents should prioritize targeted therapeutic approaches for patients with moderate-to-severe UC.

## Appendices

Appendix 1 

**Table 6:** Risk of Bias in Non Randomised Studies of Interventions (ROBINS-I) Analysis of Included Observational Studies Each domain of bias is reported per study, with overall judgment reflecting the highest risk observed among the domains. Risk of bias judgements are low, moderate, serious, critical and not reported.

Study	Confounding	Selection of Participants	Classification of Interventions	Deviation from Intervention	Missing Data	Measurement of Outcome	Selection of Reported Result	Risk of Bias
Sabhan et al. (2024) [16]	Moderate	Low	Low	Low	Moderate	Moderate	Low	Moderate
Molander et al. (2025) [17]	Moderate	Low	Low	Low	Moderate	Moderate	Low	Moderate
Amiot et al. (2020) [8]	Moderate	Low	Low	Low	Moderate	Moderate	Low	Moderate
Chaparro et al. (2021) [18]	Moderate	Low	Low	Low	Moderate	Moderate	Low	Moderate

Appendix 2 

**Table 7:** Cochrane risk of bias (ROB 2) Assessment Tool for Quality Assessment of Randomized Control Trials (RCT) Each domain is given low risk, some concern and high risk of bias scores. The overall risk of bias judgement is then made based on each domain.

Study	Randomization	Deviation in Results	Missing Data	Outcome	Reported Results	Risk of Bias
Danese et al. [3]	Low risk	Low risk	Low risk	Low risk	Some concern	Some concern
Abreu et al. [19]	Low risk	Low risk	Low risk	Low risk	Some concern	Some concern
Afif et al. [13]	Low risk	Low risk	Low risk	Low risk	Some concern	Some concern
Li et al. [20]	Low risk	Low risk	Low risk	Low risk	Some concern	Some concern

Appendix 3 

**Table 8:** Risk of Bias in Systematic Reviews (ROBIS) Tool for Quality Assessment of Systematic Review and Meta-Analysis Each domain is rated qualitatively, and the overall risk of bias reflects the highest level of concern identified across the domains. Risk of Bias judgement is given as low, high and unclear.

ROBIS Domain	Uchida et al. (2023) [9]
Study eligibility criteria	Low
Identification and Selection of Studies	Low
Data Collection and Study Appraisal	Low
Synthesis and Findings	Low
Overall Score	Low

Appendix 4 

**Table 9:** Joanna Briggs Institute (JBI) Critical Appraisal Tool for Case Report It is an 8-points assessment tool for quality appraisal of case reports

JBI Critical Appraisal Tool	Affendi et. al. (2022) [21]
Were the patient’s demographic details clearly reported?	Yes
Was the patient’s medical history clearly outlined and presented in a timeline format?	Yes
Was the patient’s clinical condition at presentation clearly described?	Yes
Were the diagnostic tests or evaluations and their results clearly detailed?	Yes
Was the treatment or intervention clearly explained?	Yes
Was the patient’s clinical status following the intervention clearly reported?	Yes
Were any adverse or unexpected events identified and described?	Partial
Does the case report offer meaningful lessons or insights?	Yes
